# Dr. Joseph O’Dwyer

**Published:** 1898-02

**Authors:** 


Dr. Joseph O'Dwyer, of New York, died at his home in that city January 7, 1898, aged 56 years. He was a native of Ohio, passed his early life in Canada and received his academic education at McGill University, Montreal. He took his doctorate degree at the College of Physicians and Surgeons at New York in 1866, and after graduation served as resident physician at the Charity Hospital (now the City hospital) and next became examining physician at Bellevue hospital. He was a member of several medical societies—city, state and national—and was ex-president of the American pediatric society. Four sons survive, his wife having died about nine years ago. Dr. O'Dwyer's memory will ever remain green for what he contributed to the relief of suffering humanity in a most modest and unassuming manner. His name will always be associated with intubation of the larynx, a method of treatment that became possible through the ingenious devices of this worthy physician. It was shown at the autopsy that his death was caused by thrombosis of the basilar artery, with softening of the right lobe of the cerebellum and in the right half of the pons, together with localised menigitis.

